# Assessment of Risk Factors for Advanced Open Angle Glaucoma Presentation among Patients Visiting Jimma University Medical Center, Jimma, Ethiopia

**DOI:** 10.4314/ejhs.v32i5.8

**Published:** 2022-09

**Authors:** Kumale Tolesa Daba, Girum W/Gebreal Gessesse, Jemmal Mohammed Molla, Tsedeke Asaminew Alemu

**Affiliations:** 1 Department of Ophthalmology, Jimma University; 2 Department of Ophthalmology, Saint Paul's Hospital Millennium Medical College

**Keywords:** Advanced glaucoma, open angle, late presentation, risk factors, Jimma University

## Abstract

**Background:**

Glaucoma is the predominant cause of irreversible blindness, particularly the late presentation. The purpose of this study is to identify the risk factors associated with late presentation in Jimma University Medical Center

**Methods:**

A case-control study was done among patients newly diagnosed to have open angle glaucoma (of any type) at Jimma University Medical Center from July 2014 – January 2019. Cases were patients/eyes diagnosed to have any type of open angle glaucoma with advanced glaucomatous disc features, whereas controls were patients diagnosed with early and moderate stages of glaucoma.

**Results:**

There were 205 (116 cases and 89 controls) participants. The mean age of the participants at the time of diagnosis was 58.3±13.4yrs. Family history of blindness, presenting IOP, type of glaucoma and age were independently associated with late presentation. Patients with family history of blindness had late advanced glaucoma five times higher than those with no family history of blindness. The presence of late glaucoma among patients with presenting intra ocular pressure < 30mmHg is lower than those having ≥30mmHg (Adjusted Odds Ratio= 0.136). Primary open-angle glaucoma patients were less likely to present with advanced glaucoma than pseudoexfoliative glaucoma patients (Adjusted Odds Ratio=0.39). The chance of presenting with late glaucoma was increased by 3.4% for every one year increment of age.

**Conclusions:**

Presence of family history of blindness, high presenting intraocular pressure, pseudoexfoliative glaucoma and old age are risk factors for late presentation of glaucoma.

## Introduction

Globally, glaucoma is one of the public health concerns affecting many people and the predominant cause of irreversible blindness (1–2). In Africa , glaucoma contributes 15% of blindness (2). Among sub-Saharan Africans, it is the second leading cause of blindness, and it is aggressive compared to Caucasians due to it's early onset and rapid progression (3). In Ethiopia, glaucoma is the fourth cause of blindness by accounting 5.2% of blindness (3). Blindness due to glaucoma in Jimma zone, Ethiopia was 9.5% (4).

There is no population based study related to the advanced stage of glaucoma. But many clinical studies showed, significant proportion of patients appeared with advanced stage of glaucoma(5). Likewise in Menilik hospital, Ethiopia, 366 (61%) patients presented with advanced stage (CDR ≥ 9.0) (6). In a study done at Jimma university hospital, Ethiopia, 31.9% of patients had advanced glaucoma in both eyes at presentation and 60% of patients had advanced glaucoma in one eye (7).

Several researches revealed that one of the main reason why glaucoma leads to blindness is late presentation or delayed attendance to health care after advanced stage of the disease has already occurred or established (7). Even though a large portion of patients present late at advanced stage of the disease, as the clinical studies showed above, there are few studies done related to risk factors for late presentation in Africa, specifically in Ethiopia. To the authors' knowledge, there is no study on this specific topic in Ethiopia regarding reasons for advanced presentation. The aim of our study is to identify the risk factors associated with presentation at advanced stage of open angle glaucoma in Jimma University Medical Center.

## Methods

All open angle glaucoma patients who came to Jimma University Medical Center, department of ophthalmology (JUDO) from July 2014 – January 2019 were included in the study. Patients aged 18 years and above diagnosed with open angle glaucoma (Primary Open Angle Glaucoma (POAG), Pseudoexfoliative Glaucoma (PXG), Normotensive Glaucoma (NTG)) for the first time were identified consecutively and grouped as case and control as per their definitions.

Cases were defined as any eyes diagnosed to have any type open angle glaucoma with advanced glaucomatous disc features having vertical cup disc ratio (C/D) >= 0.9 and/or severe Visual Field (VF) defect with Frequency Doubling Technology (FDT) test. **Controls** were patients diagnosed newly to have any type of open angle glaucoma with glaucomatous disc features vertical C/D 0.5–0.85) and / or mild or moderate VF defect with FDT test.

Patients who have been diagnosed to have glaucoma or ocular hypertension previously (history based) including having anti-glaucoma treatment or clinical evidence suggesting prior glaucoma surgery (laser or incision), angle closure glaucomas, uveitic, neovascular, traumatic, lens induced glaucoma were excluded from the study. Patients who had significant mental, hearing or speech problem were also excluded.

Thus, 116 cases and 89 controls were selected consecutively and included in the study. The sample size was determined using Epi Info (TM) version 3. 5. 1 software package, by considering the odds of advanced glaucoma in old age (70–90years) present in 13.8% of controls and odds ratio of 5.0 (8). Detecting power of 80% with statistically significant at the 5% level (alpha = 0.05), and with case to control ratio of 1:1 were considered.

A semi-structured questionnaire was developed after reviewing different literatures for the purpose of the study. The questionnaire contained socio-demographic and socio-economic characteristics of the participants, ocular characteristics, and ophthalmic and medical history. Snellen E- chart, slit lump biomicroscope, applanation tonometer, Goldman goniolens, +90 D fundus lens, FDT machine were used to collect the data.

As normal patient care follow up in JUDO, patients who were suspected to have glaucoma or diagnosed as glaucoma patients in general outpatient department, were transferred or linked to glaucoma clinic. In glaucoma clinic, these linked patients were examined in detailed again by a glaucoma specialist for confirmation and follow up purpose. Angle structure evaluation (gonioscopy), IOP measurement with Goldman applanation tonometer, dilated posterior segment examination with +90D lens were done by glaucoma specialist. Visual field testing was done using FDT (frequency doubling technology) by trained ophthalmic nurses. And interpretation of FDT result and glaucoma staging was done by the glaucoma specialist. And unreliable test results were excluded after three times trial. For this study purpose, a single glaucoma specialist established the grading of the glaucoma.

Then patients with confirmed diagnosis of glaucoma and fulfilling inclusion criteria were requested to participate in the study voluntarily. Senior ophthalmology residents and the glaucoma specialist filled the ophthalmic examination part of the questionnaire, while the historical part of the questionnaire was filled by trained ophthalmic nurses and cataract surgery students with face-to-face interview in a separate room from the examination room.

Data quality was assured through training of the data collectors, pretest done and using single glaucoma specialist to establish the diagnosis.SPSS version 16.0 soft ware was used for data analysis. Means, standard deviation, and median were used to describe the sociodemographic and socio-economic characteristics of the participants. Before univariate binary logistic analysis, Chi-square test was performed to evaluate adequacy cells. Univariate Binary logistic analysis was done to identify candidate variables for multivariate binary logistic analysis. Those variables with p-value ≤ 0.25 were included in multivariate binary logistic analysis. Adjusted odds ratio of 95% of CI and p-value less than 0.05 was used to declare statically significant association between the cases and controls.

The study was done according to the declaration of Helsinki. Ethical clearance was obtained from Jimma university ethical review board and support letter from the department of ophthalmology. Verbal informed consent was taken to confirm patient approval. Regarding the management of patients, no intervention was made for the purpose of this study and the study did not affect management of the patients.

**Operational definition**

**Staging of glaucoma**: Staging of glaucoma was based on disc features classification (9) and VF defect on FDT(10)

**Early glaucoma**: Early glaucomatous disc features (C/D <0.65) and /or Early defect on FDT

**Moderate glaucoma**: Moderate glaucomatous disc features (vertical C/D 0.7–0.85) and / or moderate VF defect on FDT

**Advanced glaucoma**: Advanced glaucomatous disc features (e.g. C/D >=0.9) and/or **Severe** VF defect on FDT

**VF defect on FDT**(10)

**Normal test**: A test with no abnormal points in the 5 areas that are more central and in the 2 nonperipheral nasal areas, and with no more than 1 abnormal point with a P < 5% in the periphery

**Early defect**: One or more abnormal points in the central 5 areas and in the 2 non-peripheral nasal areas, and/or more than 1 P (probability) < 5% defect or at least 1 P < 2% defect in the periphery. A defect is considered to be early until it reaches the limits for a moderate defect.

**Moderate defect**: More than 2 P < 2% adjacent defects, and/or more than 4 non-adjacent P < 1% defects, or more than 6 non-adjacent P < 5% defects with at least 2 P < 1% abnormal points, or more than 9 non-adjacent P < 5% defects. A defect is considered to be moderate until it reaches the limits for a severe defect.

**Severe defect**: Those tests with more than 12 abnormal points with more than 6 P < 0.5% defects, and/or more than 9 P < 1% defects.

**Pseudoexfoliative glaucoma**: **PXG** will be defined as open angle glaucoma associated with characteristic exfoliation material on the pupil margin or anterior surface of the lens on dilated biomicroscopy.

**Presenting IOP/initial IOP**: IOP taken at first presentation at the clinic

**Late presentation**: glaucoma patients who present the first time to the clinic with advanced glaucoma stage

## Results

The total participants of the study were 205 (116 cases and 89 controls). There were 140(63.8%) males and 65(31.7%) females. The mean age of the study participants at the time of diagnosis was 58.3±13.4years. For the cases, the mean age was 61.7±12.58 and for the controls the mean age was 53.85±13.33years. One hundred twenty-one participants (44 controls and 77 cases) were from rural and the remaining 84 (45 controls and 39 cases) were from urban.

The median distance from the hospital where the participants travel is 60 km with a range of 8 – 99km. Regarding the ethnic background, majorities were Oromo, 114 (44 controls, 70 cases) then followed by Amhara, 27(14 controls and 13 cases). Most of the participants were Muslims, 102 (35controls and 67 cases) followed by orthodox Christians, 77 (35 controls and 42 cases). Most participants 105(34 controls and 71 cases) were illiterate. Majority of the participants 102(35controls and 67 cases) were farmers. The median income of the participants is 10,000 ETB/year with range of 7,000 - 79,000 birr/year (Table 1).

**Table 1:** Socio demographic variables of glaucoma patients attending JUDO by case-control status

Factor	Cases	Controls
Occupation		
Farmer	67	35
Merchant	6	9
civil servant	9	19
Housewife	19	13
private organization	3	3
Others	12	10
Literacy status		
Illiterate	71	34
read and write	16	16
primary school	23	16
secondary school	3	9
collage and above	3	14
Marital status		
Single	1	5
Married	106	78
Divorced	1	2
Widowed	8	4

Related to systemic and ocular characteristics, 23 (19 cases and 4 controls) participants had a Family history of blindness. Of all, 159 (68 case and 91 cases) were self referred and 25 (12 controls and 13 cases) were referred from nearby hospitals. The remaining 19 participants (10 cases and 9 controls) were referred from health center and health post. According to the verbal report, 66 patients (31 controls and 35 cases) reported past eye care visit. 23 participants (14 controls and 9 cases) had eyeglasses. Systemic illness was reported in 25 of the participants (10 controls and 15 cases). Of these, three participants had both DM and HTN (Table 2).

**Table 2:** Family history and past medical history of glaucoma patients attending JUDOby case and control

Factor	Cases	Controls
Family history of blindness		
Yes	19	4
No	97	85
Cause of blindness		
Glaucoma	1	1
Cataract	1	0
Trachoma	1	0
Smallpox	0	1
I don't know	113	87
Past eye care visiting		
Yes	35	31
No	81	58
Eye glass of use		
Yes	9	14
No	95	70
Previous ocular surgery		
Yes	8	6
No	92	74
Systemic illness		
DM	1	2
HTN	8	5
Asthma	3	1
Others	2	0
None	101	79
HTN & DM	1	2

From the study participants 25 (14 controls and 11 cases) had information about glaucoma. At the time of diagnosis, Pseudoexfoliative glaucoma was the main diagnosis of 106 participants (24 controls and 82 cases)s. Primary Open Angle Glaucoma was seen in 65 participants (35 controls and 30 cases) (Table 3).

**Table 3:** Glaucoma related factors of glaucoma patients attending JUDOby case and control

Factors	Cases	Controls
Information about glaucoma		
Yes	11	14
No	105	75
Presenting(initial) IOP		
≥30mmHg	92	22
<30mmHg	24	67
Type of glaucoma		
POAG	30	35
PXG	82	24
NTG	4	30

Before proceeding to multivariate logistic regression to identify determinant of late presentation of glaucoma, bivariate binary logistic analysis and Chi-square test of independence were done. In Chi-square independence test, Source of referral, causes of family's blindness and marital status failed to fulfill Chi-square independence test assumption or showed cell inadequacy. So these three variables are not included in multivariate logistic regression. In bivariate binary logistic analysis, variables that had a p-value greater than 0.25 were not included in multivariate logistic regression. So, sex (p = 0.4), religion (p =0.266), and previous eye care visit (p = 0.48) were not the candidates for multivariate binary logistic regression.

The remaining variables: age, address, income, occupation, ethnic group, literacy status, distance from hospital, systemic illness, family history of blindness, glaucoma awareness, initial IOP and type of glaucoma were entered in the multivariate logistic regression. In the multiple logistic analyses by backward step-wise method, Family history of blindness, Presenting IOP, Type of glaucoma and age were found independently associated with late presentation of glaucoma (see table 4). Patients with family history of blindness had late advanced glaucoma about five times higher than those who had no family history of blindness. Late presentation among patients with presenting (initial) IOP < 30mmHg is lower than those with IOP ≥30mmHg (AOR= 0.136). The chance of presenting with late glaucoma was increased by 3.4% for every one year increment of age. Primary open-angle glaucoma patients were less likely to present with advanced glaucoma than pseudoexfoliative glaucoma patients (AOR= 0.39). Late presentation of glaucoma among patients with NTG is lower as compared to patients with Pseudoexfoliative glaucoma (AOR= 0.15) (Table 4).

**Table 4:** determinants of late presentation of glaucoma in patients attending JUDO

Variables	No of cases	No. of Controls	Univariate analysis			Multiple logistic regression		

			OR	95% CI	P-value	OR	95% of CI	P-value
Family history of blindness								
Yes	18	4	3.9	1.27, 11.98	0.017	5.13	1.417, 18.586	0.01
No	98	85	1			1		
Presenting (initial) IOP								
≥30mmHg	92	22	1			1		
< 30mmHg	24	67	0.086	0.04, 0.16	0.044	0.136	0.063, 0.293	0.00
Type of glaucoma								
POAG	30	35	0.25	0.13, 0.49	0.00	0.39	0.173, 0.881	0.023
PXG	82	24	1			1		
NTG	4	30	0.04	0.01, 0.12	0.00	0.154	0.041, 0.584	0.006
Age			1.05	1.02,1.07	0.00	1.034	1.003, 1.067	0.034

## Discussion

Worldwide in many hospital based studies, the proportions of advanced glaucoma have been studied. These studies revealed significant portion of the patients were presented with late stage or advance stage. So far a lot of researches indicated that late medical attention at the advanced stage of the disease is the main risk factor for progression to blindness among glaucoma patients (11–13).

Our study result showed that age and advanced glaucoma had relationship. According to this study, every one year increment of age leads to chance of developing advanced glaucoma by 3.4%. This might be due to the high prevalence and incidence of glaucoma among old age (14). Other reason could be delayed visiting for ophthalmic examination among old due to economical dependence, difficulties with mobility and social isolation (15,16). In addition to these reasons, even though they visited clinic, glaucoma would be missed due to lack of comprehensive eye examination, either due to lack of examination instrument or little visualization of the posterior segment related to media opacity (cataract or corneal opacity) (17,18). This finding is consistence with studies in Ghana, England and America (8,11,19).

IOP is the only modifiable risk factor for glaucoma management. Many studies showed, there is strong association between high IOP and advanced glaucoma (20,21). In this study, the odds of attending with advanced glaucoma among patients with presenting IOP less than 30mmHg was less compared to those with IOP ≥ 30mmHg, while other factors were controlled. It is obvious that most of the time, high IOP damages the optic disc rapidly. This finding was similar to the study findings in Ghana and England (8,11).

According to this study, the type of glaucoma has a significant association with late presentation of glaucoma. Late presentation of glaucoma among patients with POAG is estimated to be lower as compared to patients with Pseudoexfoliative glaucoma. And late presentation of glaucoma among patients with NTG is estimated to be lower as compared to patients with Pseudoexfoliative glaucoma. This might be due to the fact that PXG is usually unilateral which makes it difficult for early recognition from patient side. The other reason might be; the more aggressive nature of PXG which is also commonly associated with cataract that obscure visualization of posterior segment or that leads to in-comprehensive eye examination (22). In addition to the above stated reasons, pseudoexfoliative is common in the study area (6,7).

Many researches showed the risk of glaucoma are family history, African Americans descent and some genetic predisposition (23,24). Our data revealed that presentation at advanced stage of glaucoma was about five times higher among patients who had family history of blindness than those with no family history. Although the cause of the blindness in the family was not known, glaucoma might be one of the causes. Though it seems contradictory, patients who had family history of blindness had information and fear about glaucoma. But they might not have ocular evaluation for different reasons: lack of eye care service around nearby, lesser affordability to eye care service and other misconceptions about blindness (25).

In conclusion, patients with family history of blindness, those with presenting (initial) IOP ≥30mmHg and with sub-type of pseudoexfoliative glaucoma are more likely to present later (at an advanced stage) than those who had no family history of blindness, those with presenting (initial) IOP < 30mmHg and with sub-type of primary open angle glaucoma, respectively. Community screening may be important for early diagnosis and treatment of glaucoma which is the number one cause of irreversible blindness. Community education may be helpful to encourage the population for regular eye check up particularly for those people with family history of blindness.
